# *TCF7L2* correlation in both insulin secretion and postprandial insulin sensitivity

**DOI:** 10.1186/s13098-018-0338-1

**Published:** 2018-04-26

**Authors:** Mari Cassol Ferreira, Maria Elizabeth Rossi da Silva, Rosa Tsuneshiro Fukui, Maria do Carmo Arruda-Marques, Rosa Ferreira dos Santos

**Affiliations:** 1 Laboratory of Medical Investigation LIM-18, Division of Endocrinology, School of Medicine, University of Sao Paulo, Av Dr Arnaldo, 455 room 3324, Sao Paulo, SP 01246903 Brazil; 2 School of Medicine of Unochapecó University, Bairro Efapi, Chapecó, SC 89809-900 Brazil

**Keywords:** Type 2 diabetes mellitus, Insulin resistance, Polymorphism, genetic, Polymorphism, single nucleotide, Glucose metabolism disorders, Hyperglycemia, postprandial

## Abstract

**Background:**

The *TCF7L2* rs7903146 variant is strongly associated with type 2 diabetes mellitus (T2DM). However, the mechanisms involved in this association remain unknown and may include extrapancreatic effects. The aim of this study was to perform a metabolic characterization of T2DM patients with and without the *TCF7L2* rs7903146 risk T allele and analyze some influences of the *TCF7L2* genotype on glucose metabolism.

**Methods:**

Patients with T2DM (*n *= 162) were genotyped for the *TCF7L2* rs7903146 single nucleotide polymorphism. Individuals with CT/TT and CC genotypes were compared regarding basal serum levels of glucose, glycosylated hemoglobin A1C, HDL, uric acid, insulin, and C-peptide. A subset of 56 individuals was evaluated during a 500-calorie mixed-meal test with measurements of glucose, insulin, proinsulin, C-peptide and glucagon. Additional secondary assessments included determination of insulinogenic index (IGI_30_), and insulin sensitivity (%S) and resistance (IR) by Homeostatic model assessment (HOMA).

**Results:**

Patients with the CT/TT genotype showed lower baseline plasma concentrations of C-peptide when compared with those with the CC genotype. Of the 56 individuals who participated in the mixed-meal test, 26 and 30 had the CC and CT/TT genotypes, respectively. CT/TT subjects, compared with CC individuals, had higher post prandial plasma levels of insulin and C-peptide at 30–120 min (p < 0.05) and proinsulin at 45–240 min (p < 0.05). Interestingly CT/TT individuals presented at baseline higher %S (p = 0.021), and lower IR (p = 0.020) than CC individuals. No significant differences in IGI_30_ values were observed between groups.

**Conclusions:**

The T2DM individuals carrying the rs7903146 T allele of the *TCF7L2* gene presented higher IR pattern in response to a mix-meal test, different of beta cell function at baseline assessed by C-peptide levels which was lower, and Homa-IR was lower when comparing with non-carriers.

## Background

Type 2 diabetes (T2DM) is a result of a complex interplay between genetic and environmental factors interfering with glucose and fat metabolism. The disease involves multiple defects in peripheral (muscle) and hepatic insulin action, insulin secretion, adipose tissue metabolism, whole-body lipolysis, and possibly a range of additional metabolic defects in several other organs [1]. Modern genetic analysis is unquestionably one of the most powerful research tools in complex diseases such as diabetes. It has been estimated that 30–70% of the risk of development of T2DM may be associated with genetic factors [2].

About 40% of the diabetic subjects with fasting glucose levels above 120 mg/dL and 90% of those with glycosylated hemoglobin A1C (HbA1c) above 7% have abnormal postprandial glucose excursions [3]. Postprandial hyperglycemia has an estimated 30% contribution to the overall HbA1c concentration, and HbA1c levels, in turn, are proportional to the time in which the body remains in a postprandial state [3]. Even though T2DM is a polygenic disease with a wide range of phenotypic expression, the knowledge of the genetic mechanisms involved in the development and manifestations of the disease is one of the best weapons to implement therapeutic and preventive measures.

Tracking of genetic polymorphisms (i.e., single nucleotide differences between individuals) has identified single nucleotide polymorphisms (SNP) in the transcription factor 7-like 2 *(TCF7L2*) gene located in chromosome 10q25.2–10q25.3 with strong association with T2DM and impaired insulin secretion. This is a fundamental breakthrough in the understanding of the genetics in T2DM [4, 5]. An important meta-analysis has associated one such SNP, the *TCF7L2* rs7903146 variant, with an allelic odds ratio of 1.46 of T2DM development, making this the strongest known genetic risk factor for the disease [6]. *TCF7L2* is mostly expressed in adipose tissues (visceral and subcutaneous), followed by liver, pancreatic islets, brain and other tissues [7, 8]. In contrast, *TCF7L2* expression in the skeletal muscle is very low compared with other tissues [9]. The impact on diabetes of the rs7903146 variant of the *TCF7L2* or other variants of the gene associated with T2DM appears to be mediated by decreased insulin secretion associated or not with defects in insulin processing [4, 5, 10–12], reduced effects of glucagon-like peptide-1 (GLP-1) [13, 14], increased hepatic glucose production [10, 11, 13], and insulin resistance [8, 15]. This occurs because *TCF7L2* is expressed in a broad spatial domain, including tissues with important roles in glucose metabolism. This raises the possibility that *TCF7L2* may not regulate the glucose metabolism in vivo primarily through actions on beta cells, but rather through actions on extrapancreatic tissues which remain poorly characterized [15].

In this study, we performed a detailed metabolic characterization of adults with T2DM with and without the *TCF7L2* rs7903146 risk T allele. In addition, we investigated the profiles of plasma insulin, proinsulin, C-peptide, glucagon, GLP-1 and plasma glucose during a 4-h meal test to explore some influences of the *TCF7L2* genotype on glucose metabolism.

## Methods

Blood for DNA extraction and biochemical and hormonal assessments was collected from 162 individuals diagnosed with T2DM. The study was approved by the Ethics Committee of the HC-FMUSP (CAPPesq0261/09) at Universidade de São Paulo and was performed according to the Declaration of Helsinki. All subjects gave written informed consent to participate and for publication data.

We selected 182 individuals with T2DM with disease duration < 10 years, body mass index (BMI) between 25 and 35 kg/m^2^, aged 45–65 years, and nonusers of insulin, DPP-4 inhibitors, or GLP-1 mimetics. The participants were recruited from the endocrinology outpatient clinic at HC-FMUSP and from campaigns celebrating the World Diabetes Day. After the exclusion of patients with nephropathy, liver disease, and severe heart disease, 162 patients remained in the study and a subset of 56 individuals was paired according to genotype CC versus CT/TT and phenotypic variables (diabetes duration, BMI, and age) were selected for a mixed-meal test.

The individuals have had DM2 for 5.6 ± 3.5 years and without previous treatment with insulin, dipeptidyl peptidase 4 (DPP-4) inhibitors, or GLP-1 agonists. Subjects had a mean age of 57.4 ± 7.1 years, body mass index (BMI) of 30.5 ± 5.2 kg/m^2^, and mean HbA1c of 7.6 ± 1.79%. The participants were in good health, as determined by their medical histories, physical examinations, and screening blood tests. All subjects were genotyped for the *TCF7L2* polymorphic variant rs7903146. Levels of blood glucose, HbA1c, HDL, uric acid, insulin, and C-peptide were compared in carriers and non-carriers of the risk T allele (CT/TT and CC genotypes, respectively). Treatment of diabetes included metformin alone or in association with a sulfonylurea.

A subset of 56 patients, 34 women, was paired according to phenotypic variables (diabetes duration, BMI 25–35 kg/m^2^, and ages 45–55 or 56–65 years old) were selected for a mixed-meal test, which consisted of a 500-calorie breakfast comprised of 50% carbohydrates, 30% protein, and 20% fat. Sulfonylureas were discontinued for 24 h before the test. Blood samples were collected at time points 0 (or baseline, corresponding to the start of the meal) and after 15, 30, 45, 60, 90, 120, 180, and 240 min for measurement of glucose, insulin, proinsulin, C-peptide, glucagon and GLP-1. Baseline measurements also included levels of TNF-α, interleukin-6 (IL-6), C-reactive protein (CRP), and HbA1c.

For measurement of blood glucose levels, we used an enzymatic colorimetric method (system glucose oxidase–peroxidase, GOD-ANA Lab test, Brazil). Plasmatic concentrations of insulin, proinsulin, C-peptide, and glucagon were determined by double-antibody, liquid phase radioimmunoassay (Millipore Corporation, Billerica, USA). Plasma concentrations of active GLP-1 were measured with an ELISA kit (Millipore Corporation, Billerica, USA). We used the homeostasis model assessment (HOMA) calculator (available at https://www.dtu.ox.ac.uk/homacalculator/) to estimate the degree of beta cell function (%B), and insulin sensitivity (%S) and resistance (IR, which is the reciprocal of S% [100/%S]) in the overall cohort using baseline glucose and C-peptide levels. Beta cell function was also evaluated with the insulinogenic index (or insulin secretion index, IGI_30_) using the early postprandial insulin response. The index is calculated by the increase in plasma insulin level 30 min after the meal [(insulin at 30 min − fasting insulin)/(glucose at 30 min − fasting glucose)].

### Genotyping of *TCF7L2* rs7903146

Blood samples were obtained from each subject, and genomic DNA was extracted from peripheral blood leukocytes using the salting-out method. The rs7903146 SNP was genotyped using TaqMan SNP Genotyping Assays (Applied Biosystems, Foster City, CA, USA) according to the manufacturer’s instructions. The genotyping success rate was 96.4%. Direct sequencing of 10 samples with different genotypes was performed for quality control of the assays and the concordance between assays was 100%.

### Statistical analysis

Qualitative characteristics of the patients were described according to each genotype, and their association with the genotypes was assessed with the Chi square test. Laboratory assessments and anthropometric characteristics were described according to each genotype and compared among the genotypes with analysis of variance (ANOVA) or the Kruskal–Wallis test. For the rs7903146 SNP, we evaluated the genotypes CC, CT, and TT separately, and then the CT and TT in combination. The categories were then compared using Student’s *t* test or the Mann–Whitney U test, further, in Tables 1 and 2 footnote.

**Table 1:** Laboratory characteristics of the overall cohort (*n *= 162) and results of the comparative tests

Variable	rs7903146	Mean	SD	N	p
Age (years)	CC	58.42	6.15	67	0.054
	CT/TT	56.26	7.96	95	
Age/diagnosis (years)	CC	53.27	6.66	67	0.047*
	CT/TT	50.88	8.47	94	
BMI (kg/m^2^)	CC	30.43	4.51	67	0.823
	CT/TT	30.61	5.44	95	
Waist (cm)	CC	103.43	11.18	67	0.804
	CT/TT	102.99	10.92	94	
Neck (cm)	CC	38.69	3.43	67	0.053
	CT/TT	37.63	3.34	93	
N/T ratio	CC	0.67	0.08	67	0.008*
	CT/TT	0.64	0.07	93	
Blood glucose (ng/dL)	CC	146.69	59.48	61	0.988
	CT/TT	146.84	58.63	91	
HbA1c (%)	CC	7.57	1.70	61	0.943
	CT/TT	7.59	1.78	87	
HDL (mg/dL)	CC	46.48	10.88	60	0.041*
	CT/TT	50.89	13.87	87	
Uric acid (mg/dL)	CC	5.93	1.78	53	0.017*
	CT/TT	5.23	1.40	83	
Insulin (µIU/mL)	CC	17.65	12.13	63	0.099
	CT/TT	14.55	10.80	90	
C-peptide (ng/mL)	CC	3.47	1.65	63	0.015*
	CT/TT	2.87	1.36	93	

All analyses were performed with Student’s *t* test, with the exception of the comparison of C-peptide levels, which was assessed with the Mann–Whitney test

*SD* standard deviation; *BMI* body mass index; *N/T ratio* neck-to-thigh ratio; *HbA1c* glycosylated hemoglobin

*p < 0.05

**Table 2:** Clinical characteristics of the subgroup of 56 patients who participated in the mixed-meal test

Variable	rs7903146	Mean	SD	Median	Minimum	Maximum	N	p
Age (years)	CC	59.65	6.07	60.50	47	69	26	0.310
	CT/TT	57.80	7.28	58.50	40	71	30	
BMI (kg/m^2^)	CC	30.29	3.45	30.02	23.7	36.9	26	0.704
	CT/TT	29.86	4.74	29.34	21.1	39.4	30	
Disease duration (years)	CC	6.31	3.90	5.00	1	13	26	0.197
	CT/TT	4.93	3.34	4.50	0	12	30	
HbA1c (%)	CC	7.79	1.86	7.50	5.4	11.8	21	0.054
	CT/TT	6.89	0.95	6.85	5.5	9	28	
Insulin (mIU/mL)	CC	15.36	8.00	13.40	4.8	30.3	24	0.483
	CT/TT	13.79	8.09	11.80	4	42	29	
C-peptide (ng/mL)	CC	3.37	1.79	2.90	0.8	7.6	25	0.154
	CT/TT	2.80	0.90	2.70	1.3	5	30	

All analyses were performed with Student’s *t* test, with the exception of the comparison of disease duration, which was assessed with the Mann–Whitney test

*SD* standard deviation; *BMI* body mass index; *HbA1c* glycosylated hemoglobin

The analyses of the meal test were conducted by groups (CC and TT/CT), time points, and evaluation of the curve (test times) using summary measures. Comparisons between groups, time points, and test times were assessed with ANOVA. For measures showing statistically significant differences, we performed multiple Bonferroni comparisons or contrasts to estimate the differences and verify in which groups or moments they occurred. In the follow-up curve, we compared the groups at each moment using Scheffé’s multiple comparisons test.

## Results

The clinical characteristics of the overall cohort are presented in Table 1. We observed that carriers of the T allele were younger at diabetes onset, which may suggest an increased susceptibility to the disease. These patients also differed from those in the CC group by presenting lower uric acid and higher HDL-cholesterol plasma levels, considered markers of peripheral insulin resistance. We also observed that carriers of the T allele had a lower neck-to-thigh ratio.

Mean C-peptide levels differed among the three genotypes of the rs7903146 variant (p = 0.040 for CC versus CT versus TT). Carriers of the rs7903146 T allele (n = 95) showed lower baseline plasma concentrations of C-peptide when compared with non-carriers of the allele. The difference was statistically greater in CC wild-type homozygous individuals compared with CT (p = 0.029) and TT (p = 0.037) carriers.

**Fig. 1:**
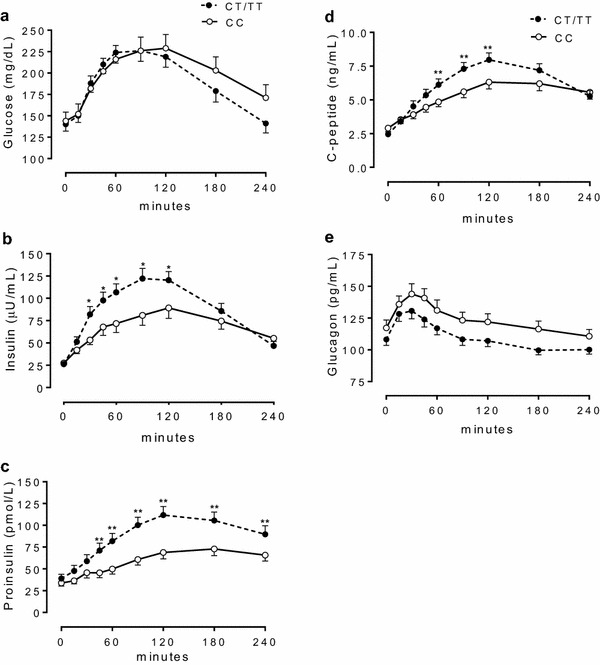
Plasma concentrations of glucose (**a**), insulin (**b**), proinsulin (**c**), C-peptide (**d**) and glucagon (**e**) in 26 and 30 carriers of the CC and CT/TT genotypes, respectively, during the mixed-meal test. CT/TT subjects, compared with CC individuals, had higher plasma levels of insulin and C-peptide at 30–120 min (p < 0.05) and proinsulin at 45–240 min (p < 0.05)

Of the 56 individuals who participated in the mixed-meal test, 26 were carriers of the CC variant, and 30 were carriers of the CT/TT variants of the rs7903146. Both groups had similar weight, BMI, and plasma concentrations of glucose and HbA1c (Table 2). Plasma concentrations of glucose, glucagon and GLP-1 exhibited similar behavior during the mixed-meal test in both the CC and CT/TT groups. In contrast, plasma concentrations of insulin during the test were significantly higher in CT/TT subjects at time points from 30 to 120 min when compared with CC individuals (p < 0.05) (Fig. 1). Insulin levels peaked at 90 min in the CT/TT group and at 120 min in the CC group. Similarly, plasma concentrations of proinsulin were also higher in CT/TT subjects, but in this case it was at almost all time points, from 45 to 240 min, compared with CC individuals (p < 0.05). Additionally, there was no significant difference in IGI_30_ in CC individuals compared with CT/TT individuals (1.52 ± 3.49 versus 1.31 ± 1.95, respectively, *p* = non-significant).

Using baseline glucose and C-peptide concentrations to calculate the HOMA index, carriers compared with non-carriers of the T allele had higher %S (p = 0.021), lower IR (p = 0.020), and similar %B (p = 0.095). There was no difference in baseline levels of CRP and IL-6 in both genotype groups, but TNF-α values were higher in the CT/TT group when compared with the CC group (p < 0.001).

## Discussion

The results of our study demonstrate that the presence of the rs7903146 T allele in the *TCF7L2* gene in individuals with T2DM was associated with increased postprandial secretion of insulin, proinsulin, and C-peptide when compared with the absence of the allele. In contrast, the serum concentrations of glucagon and glucose in response to the meal test were similar in all individuals regardless of genotype. These results show that beta cells respond differently to a glucose overload in a mixed meal when carriers of the rs7903146 CT and TT variants are compared with carriers of the CC genotype. However, the results of the analyses performed after the meal also suggest that carriers of the CT/TT variants may also show greater insulin resistance. This contrasts with the results of the HOMA analysis, which used baseline values, reflecting the limitations of using HOMA and the importance of performing a postprandial analysis. And also reinforce the likely role of TCF7L2 in incretin effect that occurs in the postprandial state. These results should be interpreted considering an absence of differences in weight or glucotoxicity in the individuals included in our study since both groups had similar BMI and plasma concentrations of glucose, glucagon, and HbA1c. Of note, GLP-1 is secreted with a magnitude proportional to the caloric content of the meal. Although carbohydrates seem to be the strongest triggers of GLP-1 secretion, ingested fat also reliably stimulates GLP-1 release [16]. In contrast, dietary proteins have a variable effect on incretin release [17].

Most of previous studies assessing the impact of *TCF7L2* SNP variants in the pathogenesis of T2DM have focused on the role of the gene in beta cells. However, some studies have demonstrated a negative effect of the *TCF7L2* rs7903146 T allele on insulin sensitivity, showing that carriers of the T allele may present reduced insulin sensitivity [18, 19]. Moreover, it has been recently demonstrated that mice overexpressing *TCF7L2* display reciprocal phenotypes, including increased plasma insulin levels, and glucose intolerance due to peripheral insulin resistance, indicating that overexpression of *TCF7L2* leads to a phenotype of T2DM [8]. In our study, we found no differences in IGI_30_ after the mixed-meal test. However, there was a difference in plasma concentrations of insulin and C-peptide at time points 30–120 min in the curve, which corresponds to moments with higher insulin levels in T allele carriers. Additionally, a study using a genetic approach in mice showed that removal of the *TCF7L2* gene from beta cells did not affect their function, whereas manipulating *TCF7L2* levels in the liver had major effects on metabolism [20]. These authors also showed that adult mice with knockout of the liver-specific *TCF7L2* gene have reduced production of hepatic glucose during fasting and improved glucose homeostasis when maintained on a high-fat diet [20].

The results of our study show that carriers of the rs7903146 T allele of the *TCF7L2* gene present lower baseline plasma concentrations of C-peptide than non-carriers, with similar plasma concentrations of glucose and glucagon. These data are in line with those of other studies that suggest that the diabetogenic effect associated with *TCF7L2* may be expressed on insulin secretion [4, 5, 18]. However, we could not find lower insulin secretion in T allele carriers when we evaluated the IGI_30_. Grant et al. [21] suggested that variants in *TCF7L2* affect an individual’s susceptibility to T2DM through impaired transcriptional regulation of the insulinotropic hormone GLP1-1, encoded by *GCG* and expressed in the brain and gut. An alternative hypothesis to explain our findings is that variants in *TCF7L2* disrupt adipogenesis and/or adipocyte function by altering the transcriptional regulation of *PPARG* leading to deposition of triglycerides in peripheral tissues (i.e., liver and muscle) and resulting in insulin resistance or that the defect on insulin secretion would be exacerbated by acute free fatty acid (FFA)-induced insulin resistance [19, 21, 22]. Although this study did not aim at evaluating peripheral insulin sensitivity in carriers of the T allele, the results suggest that further investigation should be made in this regard.

We would like to point out the relatively small number of participants (n = 56) who underwent to the mix-meal test as a limitations of our study, there is a potential contribution of this for not finding statistical differences on some of the variables. And another limitation of the study is that other TCF7L2 SNPs with more weak association with T2DM were not studied, e.g., rs7901695, rs7896340, rs11196205, and rs12255372, they could have functional impacts.

## Conclusions

We conclude that T2DM individuals carrying the rs7903146 T allele of the *TCF7L2* gene have different patterns of response at baseline and to a 500-calorie meal test, suggesting at baseline a worse quality function of the beta cells. After meal test we verified increased postprandial secretion of insulin, proinsulin, and C-peptide in the rs7903146 T allele carriers when compared with the absence of the allele. These data support that *TCF7L2* plays key roles in glucose metabolism through actions beyond those of the pancreatic beta cells and point out to functionally opposing cell-type specific effects for *TCF7L2* on maintaining a balanced glucose metabolism. These findings reinforce the role of TCF7L2 in non-pancreatic tissues, and are consistent with our hypotheses of the occurrence of correlation between *TCF7L2* in both insulin secretion and postprandial insulin sensitivity. Uncovering of these roles may lead to new therapeutic targets for T2DM.
